# (Fe,Ni)_2_P allabogdanite can be an ambient pressure phase in iron meteorites

**DOI:** 10.1038/s41598-020-66039-0

**Published:** 2020-06-02

**Authors:** Konstantin D. Litasov, Tatyana B. Bekker, Nursultan E. Sagatov, Pavel N. Gavryushkin, Pavel G. Krinitsyn, Konstantin E. Kuper

**Affiliations:** 1 Vereshchagin Institute for High Pressure Physics RAS, Troitsk, Moscow, 108840 Russia; 2 Sobolev Institute of Geology and Mineralogy SB RAS, Novosibirsk, 630090 Russia; 3 Novosibirsk State University, Novosibirsk, 630090 Russia; 4 Novosibirsk State University of Architecture, Design and Arts, Novosibirsk, 630099 Russia; 5 Budker Institute of Nuclear Physics, Siberian Branch Russian Academy of Sciences, Novosibirsk, 630090 Russia

**Keywords:** Meteoritics, Mineralogy

## Abstract

An orthorhombic modification of (Fe,Ni)_2_P, allabogdanite, found in iron meteorites was considered to be thermodynamically stable at pressures above 8 GPa and temperatures of 1673 K according to the results of recent static high-pressure and high-temperature experiments. A hexagonal polymorphic modification of (Fe,Ni)_2_P, barringerite, was considered to be stable at ambient conditions. Experimental investigation through the solid-state synthesis supported by *ab initio* calculations was carried out to clarify the stability fields of (Fe,Ni)_2_P polymorphs. Both experimental and theoretical studies show that Fe_2_P-allabogdanite is a low-temperature phase stable at ambient conditions up to a temperature of at least 773 K and, therefore, is not necessarily associated with high pressures. This is consistent with the textural relationships of allabogdanite in iron meteorites.

## Introduction

Fe_2_P plays a substantial role in the mineralogy of iron meteorites. Two polymorphic modifications of Fe_2_P−Ni_2_P solid-solution currently known are hexagonal barringerite (C22)^1,2^ and orthorhombic allabogdanite (C23)^3,4^. Barringerite was discovered in the Ollague pallasite found in Bolivia^1^. The composition determined by electron probe microanalysis was (Fe_0.58_Ni_0.42_Co_0.003_)_1.95_P. It is isostructural with the hexagonal modification of Fe_2_P with lattice parameters *a* = 5.87(7) Å and *c* = 3.44(4) Å. In a short time after its discovery, it was shown that the Ollague meteorite is a fragment of a very large, up to several hundred kilograms, Imilac pallasite, the numerous fragments of which were found in the province of Antofagasta, Chile. It turned out that the Ollague meteorite was artificially reheated, which could cause high-temperature changes in the chemical and mineral compositions^5^. It is noteworthy that barringerite has never been observed in other fragments of the Imilac meteorite or other investigated pallasites. Some barringerite findings are associated with placers^6^, but their sources are probably cosmogenic^2^.

Detailed mineralogical and crystal-chemical characterization of terrestrial barringerite from pyrometamorphic rocks of the Hatrurim formation, Israel is reported by Britvin *et al*.^2^. The mineral occurs in associations of the so-called «paralavas» − initially silicate-carbonate sedimentary rocks that remelted during pyrometamorphic processes at about 1300 K, but under low pressure. Barringerite from the Hatrurim formation is almost pure Fe_2_P, the exact composition is (Fe_1.95_Ni_0.03_Cr_0.02_)_2.00_P. The unit cell parameters determined by single crystal X-ray diffraction are *a* = 5.867(1) Å and *c* = 3.464(1) Å with Z = 3.

**Figure 1:**
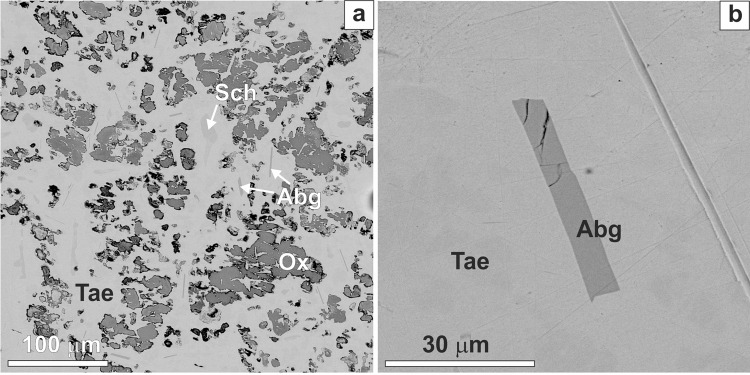
Back-scattered electron (BSE) images of the Onello iron meteorite: (**a**) Allabogdanite (Abg), schreibersite (Sch), and trevorite-magnetite oxide (Ox) phases in taenite (Tae) matrix; (**b**) enlarged area with allabogdanite crystal^10^.

The orthorhombic modification of Fe_2_P−Ni_2_P solid-solution, allabogdanite was first detected in anomalous Onello high-Ni ataxite found in 1997 in the alluvium of the Onello River, Yakutia, Russia^3^. Earlier this mineral in the Onello ataxite was considered barringerite^7–9^. The unit cell parameters of allabogdanite refined from single-crystal data are *a* = 5.792(7) Å, *b* = 3.564(4) Å, and *c* = 6.691(8) Å with *Z* = 4 and chemical composition of (Fe_1.5_Ni_0.50_Co_0.03_)_2.04_P_0.96_. A comprehensive investigation of mineralogy and trace element composition of the Onello meteorite has been carried out by Litasov *et al*.^10^. They argue that the morphology of the allabogdanite crystals and surrounding phases indicates equilibrium relationships (Fig. 1), though the Fe-Ni-P phase diagram has an intermediate Fe_3_P compound. Besides, the samples do not reveal any signatures of high-pressure modifications in the form of planar deformations, mosaicism or mylonitization.

Recently allabogdanite was identified by the electron backscatter diffraction (EBSD) in other anomalous nickeliferous ataxites, Santa Catharina found in 1875 in Brazil, and Barbianello found in 1961 in Italy^4^. Similar to the Onello meteorite, allabogdanite from Santa Catharina has a uniform chemical composition, which is (Fe_1.33_Ni_0.67_)_2.00_P. It does not show any signs of chemical zoning or interference with the host Fe-Ni matrix. The reported unit cell parameters for this allabogdanite are *a* = 5.7332(7) Å, *b* = 3.5413(6) Å, and *c* = 6.6682(10) Å. Only one allabogdanite crystal with a size of 7 μm was found in the Barbianello meteorite.

In addition, Fe_2_P is of significant importance for practical applications. It is characterized by large uniaxial magnetic anisotropy^11^, unique magnetocaloric^12,13^ and magnetoelastic^14^ properties. Therefore, intensive investigations were performed both at ambient and high pressures. The crystal structure of hexagonal modification of Fe_2_P (*P* $\bar{6}\,$2 *m*, C22-type) is described in many works^15–17^. The existence of orthorhombic Fe_2_P (*Pnma*, C23-type) was first reported in Senateur *et al*.^18^ who synthesized this modification at 8 GPa and 1073 K. Then these data were confirmed using *in situ* X-ray diffraction, where phase transition was established at 8 GPa and 1673 K^19^.

Based on the experimental results, it was concluded that allabogdanite could serve as a stishovite-grade indicator of shock metamorphism in iron meteorites^4^. To date, there are two other cases of mineralogical evidence for shock effects in iron meteorites, including (a) stishovite in IVA iron meteorite Muonionalusta^20^ and (b) high-pressure modification of apatite - tuite in IIE iron meteorite Elga^21^. In both cases, high-pressure minerals are related to shock-melt veins and deformation microstructures, which is in contrast with allabogdanite appearance (Fig. 1).

The Curie temperature of Fe_2_P is 217 K^22^. Therefore, numerous studies were devoted to increasing the Curie temperature of Fe_2_P without the reduction of the magnetic anisotropy. One of the ways is the substitution of Fe by other transition metals^23^. In (Co_*1-x*_Fe_*x*_)_2_P solid-solution, where Co_2_P is orthorhombic (*Pnma*, C23-type), hexagonal crystal structure is restricted to the relatively narrow compositional range *x* > 0.84^24^. Ellner and Mittemeijer^25^ discovered a high-temperature hexagonal modification of Co_2_P (*P* $\bar{6}\,$2 *m*, C22-type) which is stable in the temperature range from 1428 to 1659 K. The high-temperature modification of Co_2_P can be quenched to room temperature. In (Co_*1-x*_Ni_*x*_)_2_P, the orthorhombic structure is stable at *x* < 0.15^26^. Jernberg *et al*.^27^ reported hexagonal to orthorhombic transformation in partially Si-substituted Fe_2_P_*1-x*_Si_*x*_ phase. At room temperature, it takes place at *x* ≈ 0.1.

Here we would like to point out some controversial and ambiguous observations that prompted us to carry out present research. Firstly, *ab initio* calculations showed that Fe_2_P-allabogdanite is energetically favorable compared to Fe_2_P-barringerite at ambient conditions^28^, which contradicts the experimental evidence for barringerite as an ambient pressure phase^19^. Bhat *et al*.^29^ took into account the spin fluctuations to obtain preferable energetic stability of barringerite at ambient conditions, which, according to the authors, is an “exceptional case” in *ab initio* calculations. Secondly, C23 Co_2_P-allabogdanite modification is thermodynamically stable at ambient conditions^25^. In this paper, we report the results of *ab initio* calculations and experimental investigation of the stability fields of polymorphic modifications of (Fe,Ni)_2_P, which show that allabogdanite is thermodynamically stable at ambient *PT*-conditions.

## Results

### *Ab initio* calculations

**Figure 2:**
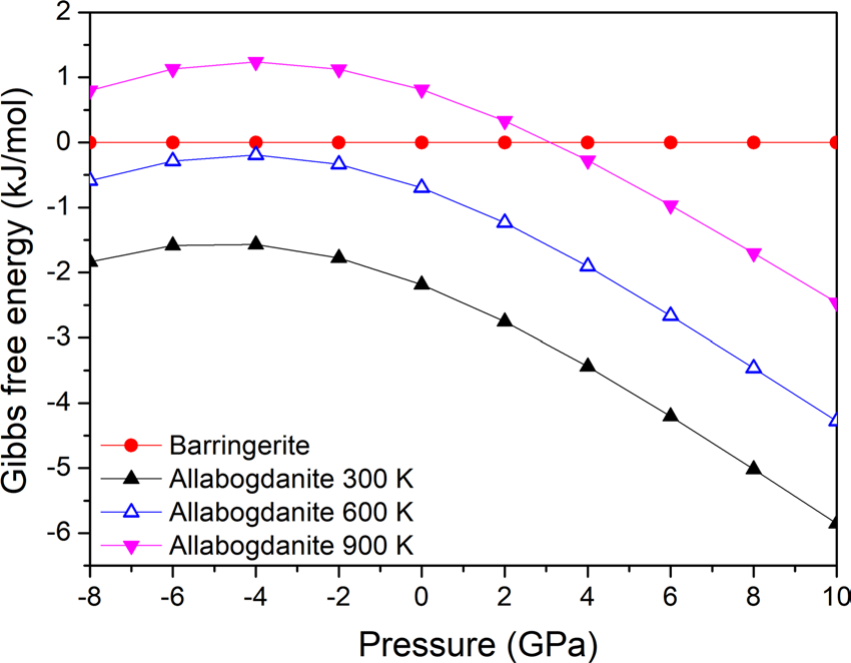
Calculated Gibbs free energy for Fe_2_P allabogdanite and barringerite modifications at 300, 600, and 900 K.

Both Fe_2_P modifications, barringerite and allabogdanite, are dynamically stable at 0.1 MPa. The calculated Gibbs energy difference between allabogdanite and barringerite at ambient pressure and 298 K is −2.19 kJ/mol (−22.6 meV/f.u.), whereas at 600 K it is −0.69 kJ/mol (−7.2 meV/f.u.) with calculation accuracy of ±0.96 kJ/mol (Fig. 2). Between 600 and 900 K, barringerite becomes a stable phase at 0.1 MPa. Our Gibbs free energy calculations for Co_2_P show that the C22 modification is dynamically unstable at 0 K and ambient pressure. Static calculations show that the C23 Co_2_P-phase has a lower enthalpy than the C22 phase (Fig. S1). Thus, for Co_2_P there is only one stable modification at atmospheric pressure, which has C23 allabogdanite crystal structure.

**Figure 3:**
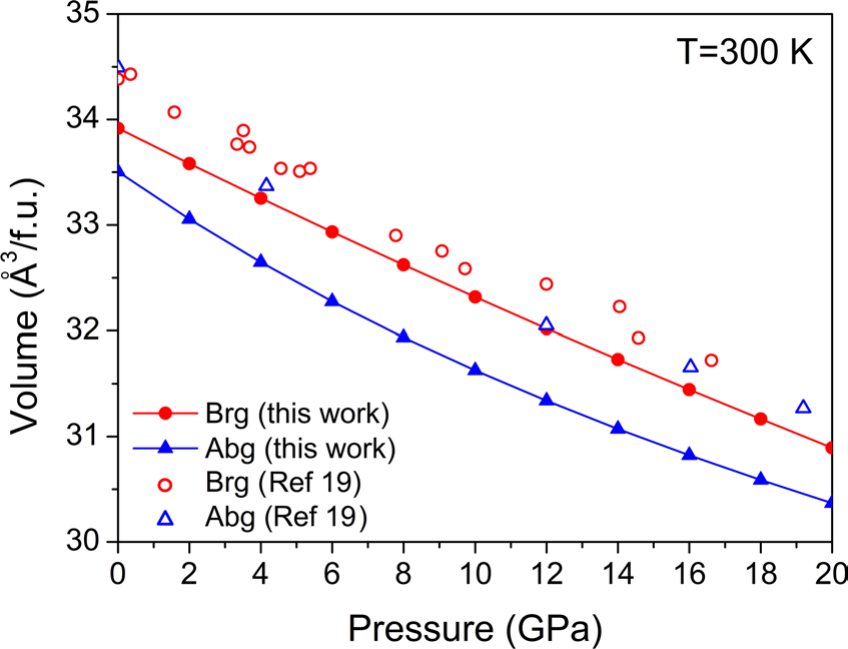
Comparison of calculated *PV*-diagram for Fe_2_P allabogdanite and barringerite at T = 300 K with experimental data^19^.

**Figure 4:**
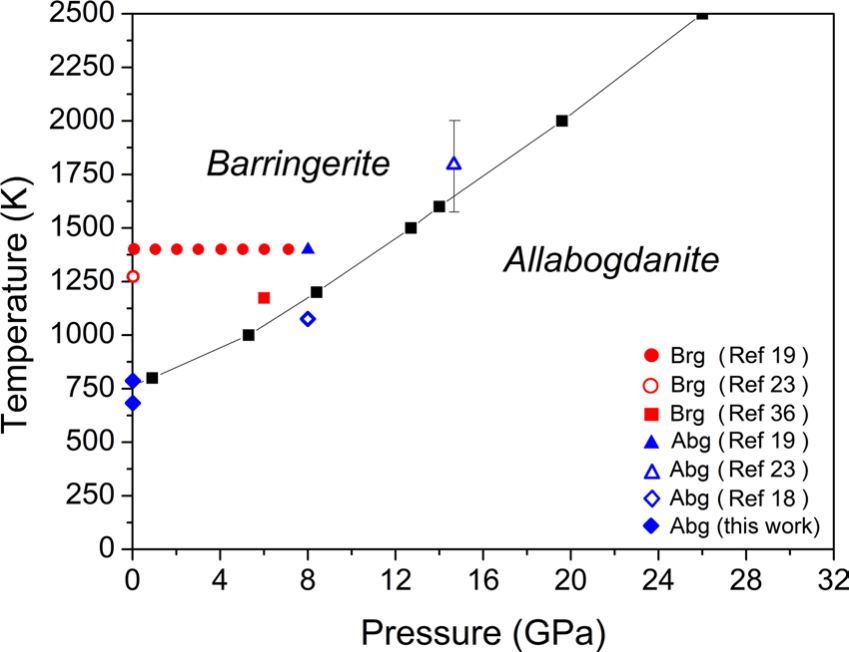
Calculated *PT*-diagram for Fe_2_P allabogdanite (Abg) and barringerite (Brg). Black line shows the phase transition boundary based on data from *ab initio* calculations.

Fe_2_P-allabogdanite has a smaller unit cell volume of 33.50 Å^3^/f.u. than Fe_2_P-barringerite (33.91 Å^3^/f.u.) at normal conditions. Similar relations are observed at high pressures up to 20 GPa (Fig. 3). In contrast, Dera *et al*.^19^ noticed that Fe_2_P-allabogdanite has marginally larger unit cell volume than Fe_2_P-barringerite (corresponding data are shown in Fig. 3), which may imply the stability of the latter at ambient conditions. The bulk moduli of the C22 and C23 phases, determined using 3rd order Birch Murnaghan EOS are 199(9) GPa (with *K*′ = 1.4) and 142(2) GPa (with *K*′ = 5.5), respectively. The constructed *PT*-diagram for Fe_2_P, based on the calculation of Gibbs free energy, shows that at ambient conditions Fe_2_P is stable in the form of allabogdanite with the transition to barringerite at ~750 K. With increasing pressure, the stability field of allabogdanite expands, and at a pressure of 25 GPa, the phase transition occurs at ~2500 K (Fig. 4).

### Experimental study of the stability fields of (Fe,Ni)2P modifications

We performed the annealing of Fe_2_P-barringerite reagent, Fe-P, and Fe-Ni-P mixtures at 673–773 K to confirm the stability of allabogdanite at atmospheric pressure. The experimental conditions, reaction products and their compositions are summarized in Table 1.

**Table 1:** Initial compositions of the samples and the results of their characterization by XRD and SEM/EDX analysis after annealing in evacuated quartz ampoules.

Starting reagents	Temperature, K/Run duration, days	Run products
Fe_2_P-Brg reagent	673 / 30	Fe_2.03(2)_P Brg Fe_1.10(1)_P Fe_3.03(6)_P
2Fe + P	673 / 30	Fe_2.00(4)_P Abg Fe
1.5 Fe + 0.5 Ni + P	673 / 45	Fe_1.6(2)_Ni_0.3(2)_P Abg Fe_0.7(3)_Ni_1.4(4)_P Brg Fe
Fe_2_P-Brg reagent	773 / 15	Fe_1.98(2)_P Brg Fe_1.12(8)_P Fe_3.03(6)_P
2Fe + P	773 / 15	Fe_1.97(4)_P Abg Fe
1.5 Fe + 0.5 Ni + P	773 / 15	Fe_1.6(2)_Ni_0.3(2)_P Abg Fe_0.4(3)_Ni_1.6(4)_P Brg Fe

**Figure 5:**
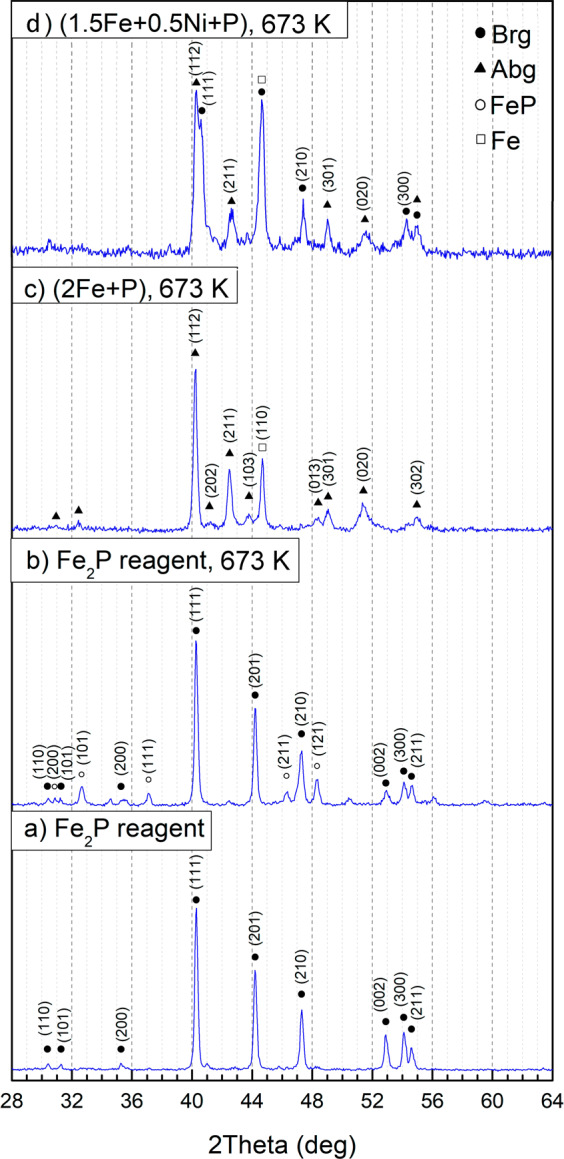
X-ray powder diffraction patterns of the samples: (**a**) reagent Fe_2_P before experiment; (**b**) reagent Fe_2_P, (**c**) mixture of Fe and red P, corresponding to Fe_2_P stoichiometry, and (**d**) mixture of Fe, Ni and red P, corresponding to Fe_1.5_Ni_0.5_P stoichiometry, after sequential annealing at 673 K for 30 days (**b,c**) and 45 days (**d**) in sealed quartz ampoules.

**Figure 6:**
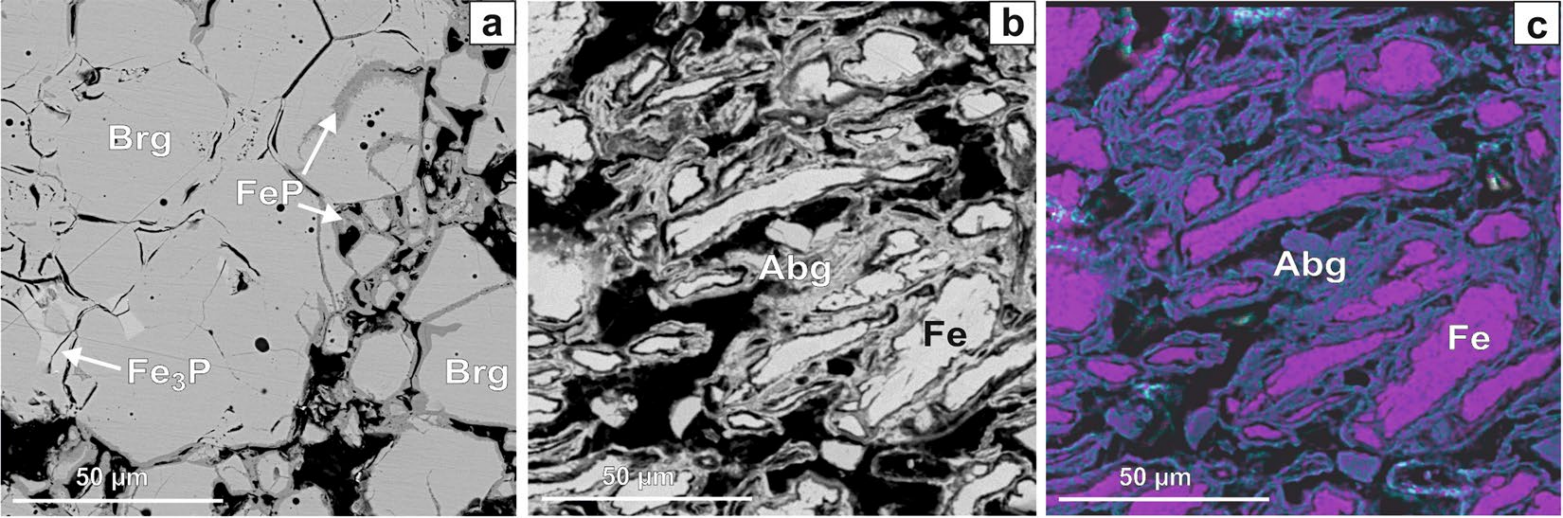
BSE image of Fe_2_P reagent (**a**); and stoichiometric Fe_2_P mixture of Fe and red P (**b**) after sequential annealing at 673 K for 30 days in sealed quartz ampoule. (**c**) elemental mapping of the area shown in (**b**). *Brg* − barringerite, *Abg* − allabogdanite. Black − pore spaces.

After annealing at 673 K for 30 days, the X-ray diffraction (XRD) pattern of the Fe_2_P reagent corresponds to barringerite modification^16^. However, additional peaks of another phase, FeP^30^, appeared (Fig. 5b). Recently, a natural analog of FeP phase, muraskoite, has been described in pyrometamorphic rocks of the Hatrurim Formation^31^. Using the scanning electron microscopy, we have determined Fe_2_P with a minor amount of FeP-murashkoite and, besides, rare grains of Fe_3_P-schreibersite (Fig. 6a). The compositions of the phases are as follows: barringerite Fe_2.03(2)_P, murashkoite Fe_1.10(1)_P, and schreibersite Fe_3.03(6)_P (Table 1). The appearance of Fe_3_P and FeP may be explained by minor heterogeneity of the starting Fe_2_P powder or reaction of Fe_2_P with the P-bearing vapor along grain boundaries during synthesis.

**Figure 7:**
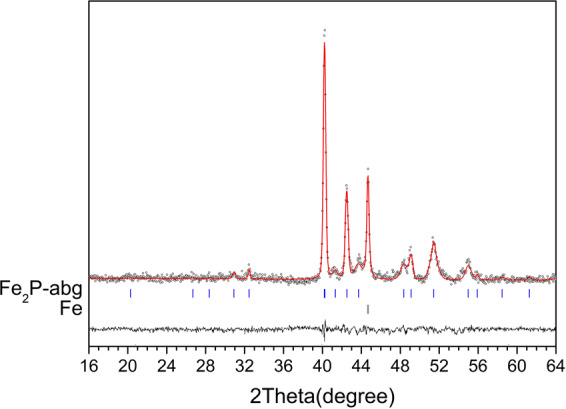
Observed (open circles) and calculated (red line) X-ray diffraction pattern of the stoichiometric mixture of Fe and red P, corresponding to Fe_2_P, after sequential annealing at 673 K for 30 days, with the difference at the same scale, plotted below. The vertical bars indicate the angular positions of the allowed Bragg reflections for allabogdanite (abg)^3^ and Fe.

The XRD pattern of the stoichiometric mixture of Fe and red P, corresponding to Fe_2_P, clearly reveals the presence of two phases, Fe_2_P-allabogdanite and α-iron (*Im*3*m*) (Fig. 5c). BSE images and elemental mapping (Fig. 6b,c) show the unreacted areas of iron grains surrounded by allabogdanite rims. The determined composition of allabogdanite is uniform, Fe_2.00(4)_P. We used whole-profile fitting to simultaneously fit both phases using the Le Bail method through the MAUD software^32^, *R*_*b*_ = 10.267% (Fig. 7). Refined allabogdanite unit cell parameters are *a* = 5.791 Å, *b* = 3.552 Å, and *c* = 6.656 Å with the unit cell volume of 136.92 Å^3^ (Table 2).

**Table 2:** The results of whole-profile fitting of X-ray diffraction patterns of synthesized phases using the Le Bail method through the MAUD software^32^.

Phase	T, K	Unit cell parameters, Å			V, Å^3^	*R*_*b*_, %
		*a*	*b*	*c*		
Fe_2_P-allabogdanite	673	5.791(3)	3.552(3)	6.651(5)	136.9(3)	10.267
Fe_2_P-allabogdanite	773	5.802(8)	3.553(5)	6.652(6)	137.0(8)	11.003
(Fe,Ni)_2_P-allabogdanite	673	5.775(3)	3.522(2)	6.646(4)	135.1(6)	10.241
(Fe,Ni)_2_P-allabogdanite	773	5.774(8)	3.522(5)	6.642(7)	135.0(7)	9.542
(Fe,Ni)_2_P-barringerite	673	5.845(2)	−	3.385(2)	100.1(3)	10.241
(Fe,Ni)_2_P-barringerite	773	5.844(3)	−	3.384(2)	100.0(4)	9.542
Fe_2_P (reagent) Brg	673	5.868(1)	−	3.4569(7)	103.0(6)	9.571
Fe_2_P (reagent) Brg	773	5.868(1)	−	3.4569(9)	103.09(8)	8.958
(Fe_1.5_Ni_0.5_Co_0.03_)_2.04_P_0.96_ Abg^3^	−	5.748(2)	3.548(1)	6.661(2)	135.8(1)	−

*R*_*b*_ – R-Bragg value for the whole refinement.

**Figure 8:**
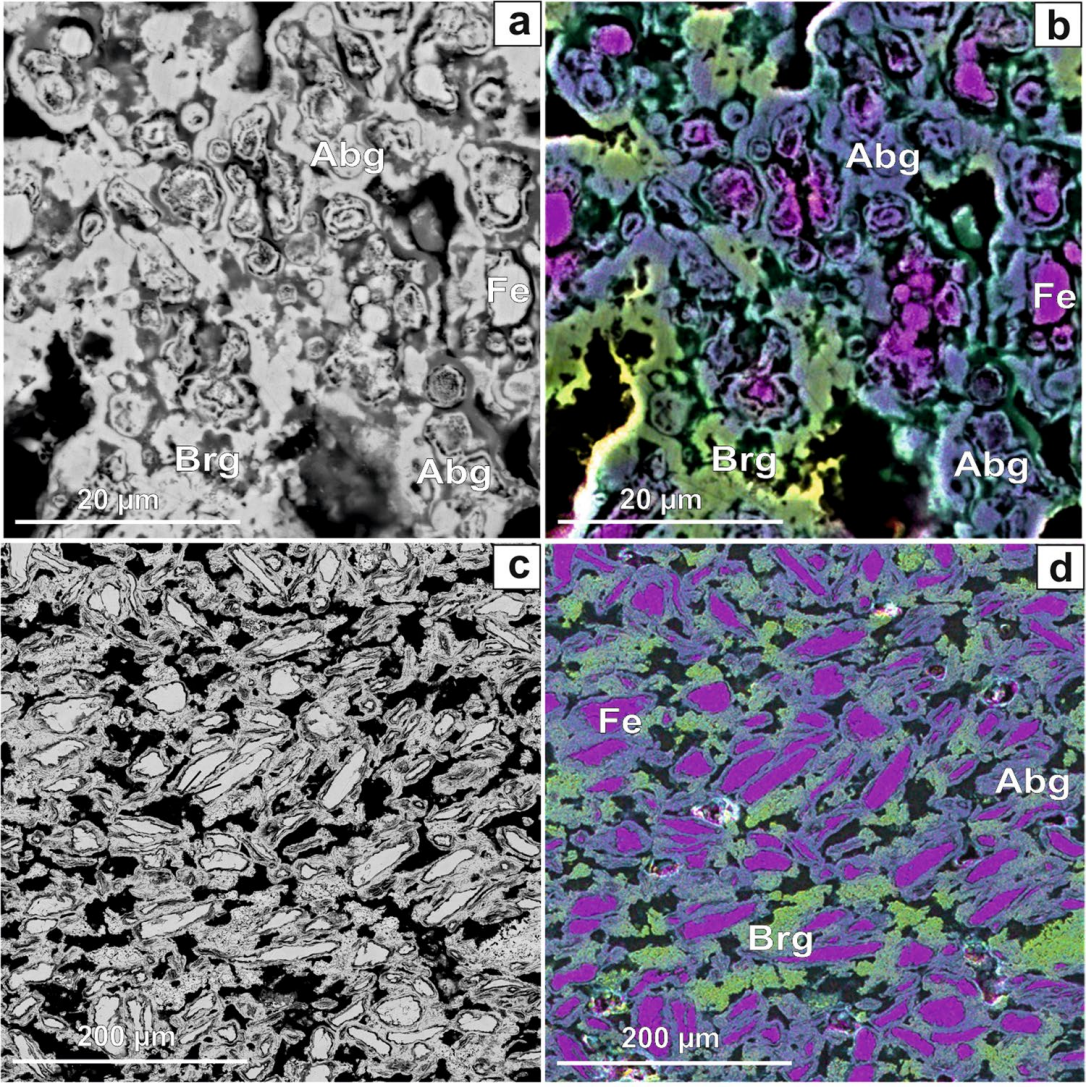
BSE image (**a,c**) and elemental maps (**b,d**) of the stoichiometric mixture of Fe, Ni and red P, corresponding to Fe_1.5_Ni_0.5_P, after sequential annealing at 673 K and 45 days (**a,b**) and 773 K and 15 days (**c,d**) in sealed quartz ampoule. *Brg* − barringerite, *Abg* − allabogdanite. Black − pore spaces.

**Figure 9:**
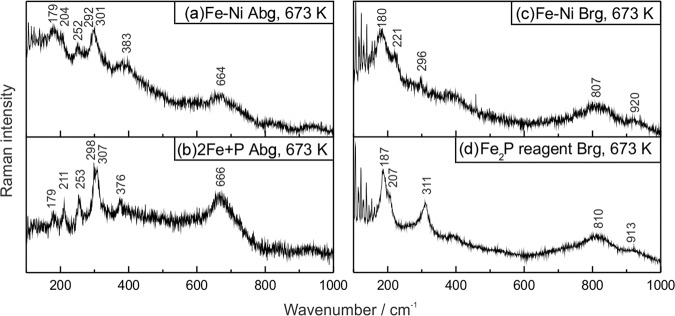
Raman spectra of allabogdanite (Abg) and barringerite (Brg). See Table 1 for sample description.

The most important results have been obtained for the stoichiometric mixture of Fe_1.5_Ni_0.5_P composition (see Table 1), which is close to that of the allabogdanite phase described in iron meteorites^3,10^. In this case, X-ray diffraction peaks of both allabogdanite and barringerite as well as unreacted Fe are observed (Fig. 5d). The composition of the phosphide varies over a broad range of concentrations, from 6 to 80 wt. % Ni, as illustrated by elemental mapping (Fig. 8a,b). The Ni-enriched areas correspond to the barringerite modification, while Fe-enriched areas, adjacent to unreacted iron grains, to the allabogdanite modification (Fig. 8b). This was confirmed by Raman spectroscopic measurements of Ni-poor and Ni-rich areas of (Fe,Ni)_2_P. The Ni-rich areas correspond to the Raman spectra of barringerite, whereas Fe-rich areas correspond to the spectra of Fe_2_P allabogdanite (Fig. 9). The reference Raman spectra were obtained for Fe_2_P allabogdanite and barringerite confirmed by XRD measurements (Fig. 9). The Raman spectra of both allabogdanite and barringerite consist of very weak lattice LO-TO vibrations at 179–376 cm^−1^ and broad presumably higher-order modes at 664 (Abg) and 807 (Brg) cm^−1^. The band positions are different from those for Fe-O and P-O vibrations in related oxide and phosphate compounds^33,34^ indicating that the modification of sample under laser beam was minimized and Raman bands correspond to the Fe_2_P compounds.

We also performed EBSD measurements to clarify the compositions of (Fe,Ni)_2_P polymorphs formed at 673 K and 773 K. In both samples, EBSD from iron grains was observed with a mean angular deviation (MAD) of 0.07–0.1° (Fig. S3). Only a few points were found for barringerite structure with a MAD of about 0.6° (Fig. S3), whereas no acceptable points were found for the allabogdanite structure. This may be because the size of the individual allabogdanite crystals is less than 1–2 microns, as well as with the inability to polish the spongy grain surface.

The determined average composition for the solid solution with allabogdanite structure at 673 K is Fe_1.6(2)_Ni_0.3(2)_P (Ni concentration varies from 6 to 28 wt.%), whereas that for barringerite structure is Fe_0.7(3)_Ni_1.4(4)_P (Ni concentration varies from 34 to 80 wt.%). The formation of the (Fe,Ni)_2_P solutions with both allabogdanite and barringerite structures can be accounted for by their different Ni/Fe concentrations. An increase in the Ni content in (Fe,Ni)_2_P solution stabilizes the barringerite modification, as Ni_2_P crystallizes only in barringerite structure^35^. It should be noted that for (Ni,Co)_2_P solution the C23/C22 phase transition occurs at about 15 mol.% Ni^26^ (Fig. S2). The equilibrium state was not achieved in our experiments. However, the results unequivocally confirm the formation of allabogdanite modification of (Fe,Ni)_2_P at atmospheric pressure.

**Figure 10:**
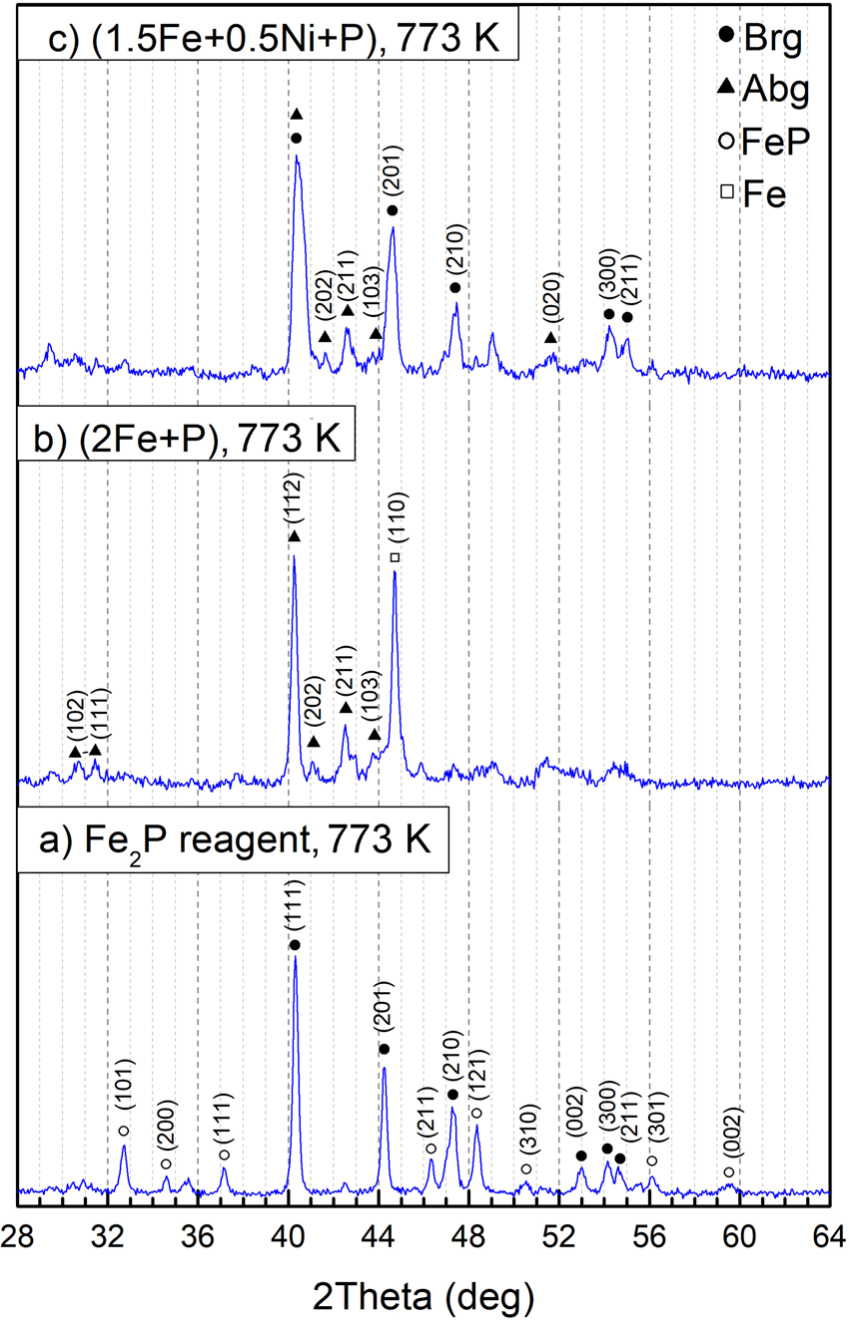
X-ray powder diffraction patterns of the samples after sequential annealing at 773 K for 15 days in sealed quartz ampoule: (**a**) reagent Fe_2_P; (**b**) stoichiometric Fe_2_P-mixture of Fe and red P, and (**c**) Fe, Ni and red P, corresponding to Fe_1.5_Ni_0.5_P.

In general, the results obtained after annealing of samples at 773 K are very similar to those annealed at 673 K. The annealed Fe_2_P reagent contains Fe_2_P-barringerite and FeP (Fig. 10a). The results of the whole-profile fitting of the X-ray profiles of samples annealed at 773 K obtained are summarized in Table 2. For comparison, we included the powder cell data on Fe_1.5_Ni_0.5_P from the Onello meteorite^3^ in Table 2. The determined unit cell volumes for (Fe,Ni)_2_P allabogdanite formed in our experiments at 673 K and 773 K are 135.1(6) Å^3^ and 135.0(7) Å^3^, respectively, which is close to that determined for (Fe_1.5_Ni_0.5_Co_0.03_)_2.04_P_0.96_ from the Onello meteorite^3^, 135.8(1) Å^3^. Minor schreibersite was also identified by scanning electron microscopy. Phase compositions are as follows: barringerite Fe_1.98(2)_P, murashkoite Fe_1.12(8)_P, and schreibersite Fe_3.03(6)_P.

Annealing of the stoichiometric Fe-P mixture leads to the formation of allabogdanite and unreacted Fe metal (Fig. 10b). Thus, it can be concluded that at a temperature of 773 K allabogdanite is still a stable modification of Fe_2_P. Allabogdanite composition is uniform and close to that determined at 673 K: Fe_1.97(4)_P. In the annealed Fe_1.5_Ni_0.5_P composition, allabogdanite, barringerite, and unreacted Fe are observed (Fig. 10c). Corresponding BSE image and elemental mapping are shown in Fig. 8c,d. The same broad range of solid solution compositions was found. The phases were also identified by Raman spectroscopy and EBSD, which gave similar results with the run at 673 K (Figs. 9 and S3).The determined average composition for the solid solution adjacent to unreacted iron grains with allabogdanite structure is Fe_1.6(2)_Ni_0.3(2)_P (Ni concentration varies from 6 to 24 wt.%), whereas that for Ni-enriched areas with barringerite structure is Fe_0.4(3)_Ni_1.6(4)_P (Ni concentration varies from 49 to 81 wt.%). The compositions of the phases are summarized in Table 1.

## Discussion

Several experimental works on the Fe_2_P phase diagram are known. Senateur *et al*.^18^ reported C22/C23 phase transition at 8 GPa and 1073 K. The indicated volume for С23 modification is smaller than that for С22 (34.24 Å^3^/f.u. and 34.36 Å^3^/f.u., respectively). The determined parameters of the phase transition and relationship between the unit cell volumes are in agreement with the results of our *ab initio* calculations (Fig. 3).

To specify the stability fields of Fe_2_P polymorphs Dera *et al*.^19^ performed *in situ* synchrotron X-ray diffraction experiments at high pressures and temperatures. Polycrystalline sample of Fe_2_P, obtained through high-temperature synthesis (the temperature of the synthesis was not indicated), was compressed in a diamond anvil cell (DAC) with laser heating to about 1400 K at each pressure step with 1 GPa interval. The authors revealed the existence of Fe_2_P barringerite at pressures from 0 to 7 GPa and Fe_2_P-allabogdanite at pressures from 8 GPa to 40 GPa and temperature of 1400 K. It was shown that allabogdanite can be recovered to ambient conditions and then, when reheated to 1400 K, transforms back to barringerite. These data provided a significant constraint on the *PT*-history of iron meteorites, which assumes: (a) the formation of allabogdanite at pressures above 8 GPa and high temperature without subsequent heating above 1400 K at lower pressures, or (b) formation of barringerite and its transformation to allabogdanite during shock metamorphism to pressures above 8 GPa and temperatures above 1400 K. The authors note that the ambient pressure density of allabogdanite is marginally lower than that of barringerite, which is inconsistent with our data in Table 2.

Gu *et al*.^23^ performed a study of Fe_2_P using Mossbauer spectroscopy in laser-heated DAC. Initial Fe_2_P-barringerite phase was synthesized at 1273 K for 3 hours in quartz tube from stoichiometric amounts of Fe and red P. Allabogdanite phase was observed at 14.5 GPa and 1800 K. Minin *et al*.^36^ investigated the Fe-Fe_2_P phase diagram at 6 GPa and showed that barringerite phase is stable from 1173 K to a congruent melting temperature of 1848 K. All experimental data at high pressures are broadly consistent with *PT*-parameters of allabogdanite to barringerite transition determined by our *ab initio* calculations (Fig. 4) indicating accuracy of DFT method applied.

We think that there are two main reasons why barringerite is erroneously considered to be a thermodynamically stable modification at ambient conditions. Firstly, this is because Fe_2_P-barringerite reagent is typically synthesized at temperatures above 1223 K, which are higher than the C23 to C22 phase transition temperature. Secondly, because barringerite is extremely persistent as a metastable modification at ambient pressure. There are two non-equivalent Fe sites in the crystal structure of both polymorphs, Fe I and Fe II. According to Mössbauer spectroscopic measurements, during the phase transition, a fall of quadrupole splitting in the Fe II site takes place, while other hyperfine parameters change insignificantly^23^. This fact indicates that the Fe II site may be a phase transition trigger. During the phase transition, half of the P atoms displace along the *c*-hexagonal axis by ~1.7 Å, preserving the topology of the Fe lattice^19^. While the total energy of the resulting crystal structures at ambient conditions is nearly equal (see Fig. 2), breaking the three Fe-P bonds, which is necessary for the mobilization of P atoms, most likely requires significant kinetic energy to surmount activation barrier. This is a reason for enhanced Fe_2_P-barringerite reagent stability at annealing at 673 for 30 days and 773 K for 15 days and ambient pressure without phase transition to a thermodynamically stable at these conditions allabogdanite modification.

Based on the results of several experimental investigations (e.g., ref. ^27^, where it is stated that C22-structured Fe_2_P is stable down to 10 K), barringerite has been considered a thermodynamically stable modification at ambient conditions. Most likely, this was the reason why in carrying out *ab initio* calculations it was barringerite that the authors attempted to stabilize. In Zhao *et al*.^37^, the stabilization of C22-barringerite phase at ambient conditions was reached with the use of the Hubbard parameter (DFT + U approach). However, the authors did not provide the details of their calculations. DFT calculations carried out by Bhat *et al*.^29^ showed that C22/C23 phase transition occurred at about 300 K indicating that C23-allabogdanite can be stable at ambient conditions. The use of DFT + U approach also led to the stability of allabogdanite^29^. Finally, considering the energy contribution of zero-point spin fluctuations (ZPSF) Fe_2_P-barringerite phase became energetically favorable, which, according to the authors, represents the exceptionally rare case of bulk material stabilization^29^. Our DFT calculations have shown that the allabogdanite phase is stable at ambient conditions without ZPSF energy contribution. Zero-point spin fluctuations play an important role at temperatures close to 0 K and are significant, for example, for superconductors^38,39^. Typically, at finite temperature, the contribution of ZPSF to the total energy is negligible in comparison with the contribution of thermal vibrations.

In this study, we suggest that (Fe,Ni)_2_P allabogdanite may be an ambient pressure phase and should not be considered as an indicator of high-pressure transformations by impact processes in iron meteorites. The observed morphology of allabogdanite grains indicates equilibrium-like relations with (Fe,Ni)-metal (Fig. 1), which is consistent with the formation at low pressures. However, the details of allabogdanite origin remain poorly constrained, because it appears in direct contact with (Fe,Ni)-metal. At the same time, there is an intermediate schreibersite-structured (Fe,Ni)_3_P phase in the Fe-P^36^ and Ni-P^40^ phase diagrams at ambient and at high pressures. The ternary Fe-Ni-P diagram has not yet been studied in the phosphorus enriched region. The Ni-enrichment of iron meteorites may be responsible for the formation of (Fe,Ni)_2_P – (Fe,Ni)-metal equilibrium as allabogdanite appears in Ni-rich ataxites only^3,4,10^. There are two alternative possibilities. First, the appearance of allabogdanite may be related to the local disequilibrium and fast entrapment of (Fe,Ni)_2_P barringerite crystals by solidifying Fe-Ni metal with subsequent very slow cooling, which allows barringerite-allabogdanite back transformation. Second, allabogdanite and (Fe,Ni)-metal can be condensed together from the gas phase without subsequent heating (above allabogdanite stability temperatures) and melting.

The results of *ab initio* calculations and experimental study indicate that allabogdanite is a thermodynamically stable polymorphic modification of (Fe,Ni)_2_P solid solution with Ni concentration of at least 20 wt.% (the value of the Ni-content in meteoritic allabogdanite) at ambient pressure and temperatures below 773 K. The exact temperature of allabogdanite-barringerite phase transition was not determined in present experiments due to the significant sublimation of red phosphorus at temperatures of about 873 K in quartz ampules and the corresponding change in the stoichiometry of the initial mixture. Hence, the temperature of phase transition and its dependence on pressure and nickel concentration is the subject of further research.

## Methods

### Sample preparation

The samples for investigation were commercially available Fe_2_P powder (99.9% Alfa Aesar) and stoichiometric mixtures of Fe (99.9%), Ni (99.9%), and red P (99.5%) corresponding to the Fe_2_P and Fe_1.5_Ni_0.5_P. The mixtures were ground under ethanol in an agate mortar, pressed into 4-mm diameter pellets with a weight of 50 mg, and sealed into a quartz ampoule under vacuum. The samples in the ampoule were separated by quartz rods.

### Experimental conditions

**Figure 11:**
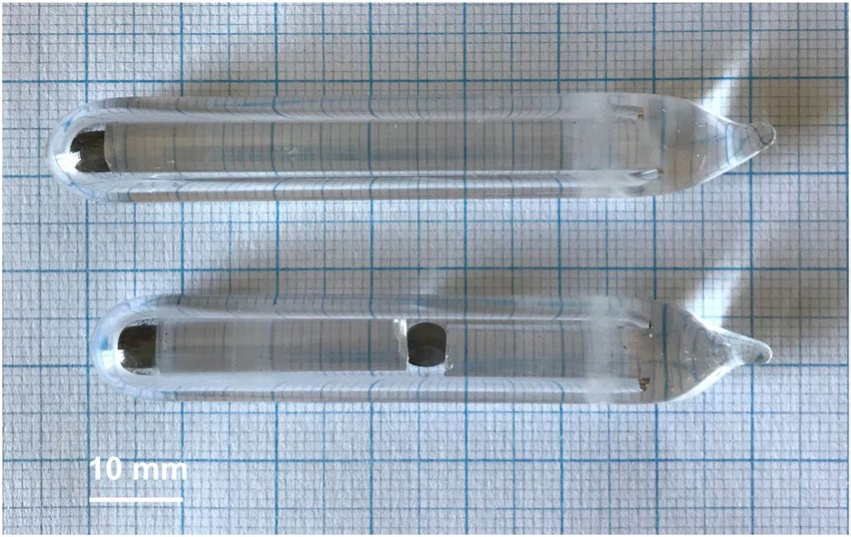
Photograph of quartz ampules with samples after sequential annealing at 773 K.

In experiment #1 all samples except Fe_1.5_Ni_0.5_P mixture were annealed at 623 K for 1 day and at 673 K for 30 days. The Fe_1.5_Ni_0.5_P mixture was annealed at 623 K for 1 day and at 673 K for 45 days (experiment #2). For experiment #3 the samples were annealed at 623 K, 673 K, and 723 K (all for 1 day), and at 773 K for 15 days (Fig. 11). The residual pressure inside the quartz ampoules was below 133 Pa.

### Scanning electron microscopy

Samples after annealing were mounted into epoxy, polished and studied using a MIRA 3 LMU scanning electron microscope (Tescan Orsay Holding) coupled with an INCA energy-dispersive X-ray microanalysis system 450 equipped with the liquid nitrogen-free Large area EDS X-Max-80 Silicon Drift Detector (Oxford Instruments Nanoanalysis Ltd) at IGM SB RAS. Energy-dispersive X-ray (EDX) spectra were collected over a 1–10 μm sample area at 20 kV accelerating voltage and 1.5 nA beam current^36^. Live counting time for X-ray spectra was 30 s. We used the same calibration technique as reported previously^36,40^.

### X-ray powder diffraction

The composition of the samples was characterized by X-ray powder diffraction (XRD) using DRON 4 (Russia) with CuKα (1.5418 Å) radiation in the 2*θ* range of 5–65°, with a step width of 0.05° and 3 s of exposure time per position at IGM SB RAS.

### Electron backscatter diffraction

Prior to the EBSD investigation, the samples were additionally polished with acidic nanometer alumina with particles of 50 nm in size. The EBSD data were collected on a Hitachi S-3400 N scanning electron microscope equipped with an Oxford Instruments HKL detector at the Siberian Synchrotron and Terahertz Radiation Centre (SSTRC), INP SB RAS. The measurement parameters include 0.5–1.0° accuracy of sample misorientations, 20 kV accelerating voltage, 8 nA beam current, 15.5 mm working distance, and 70° tilt angle. The Kikuchi pattern of each individual point was automatically indexed by the Oxford data collection software^41^.

### Raman spectroscopy

The Raman spectra were recorded on a Horiba Jobin Yvon LabRAM HR800 spectrometer with a 1024-pixel LN/CCD detector using the 532 nm wavelength of an Nd-YAG laser in a backscattering geometry using Olympus BX41 confocal microscope at IGM SB RAS^21^. The spectral resolution of the recorded Stokes side of the Raman spectrum was set to ~2.0 cm^−1^ at a Raman shift of 1300 cm^−1^. This resolution was achieved by using one grating with 1800 grooves/mm and equivalent 150-μm slits and pin hole. The microscope with an Olympus 100× objective lens of WD = 0.37 mm with 0.75 numerical aperture for visual spectral range produces a focal spot diameter of ~2 μm. The power of the laser light was set to about 0.5 mW on the sample to avoid the sample heating^21^. The spectroscopic analysis of studied phosphides was very difficult, therefore we used 50 sec duration repeated for 10 times to record one spectrum.

### Ab initio calculations

The electronic structure calculations were performed within density functional theory (DFT) implemented in the VASP 5.4 package^42,43^. The exchange-correlation effects were treated in the generalized gradient approximation (GGA) with the Perdew-Burke-Ernzerhof (PAW) scheme^44^. All calculations were performed taking into account spin-polarization with the following settings: the energy cut-off was set to 600 eV; the Monkhorst-Pack k-point mesh was 7 × 7 × 10 for Brg, and 7 × 7 × 6 for Abg; electronic smearing – with Metfessel-Paxton scheme; and σ = 0.05 eV. The temperature effect was considered within the quasi-harmonic approximation (QHA) using PHONOPY code^45^. In these calculations, the energy cut-off was set to 800 eV.

## Supplementary information

Supplementary figures.

## References

[1] Buseck PR, Goldstein JI (1969). Olivine compositions and cooling rates of pallasitic meteorites. Geol. Soc. Am. Bull..

[2] Britvin SN, Murashko MN, Vapnik E, Polekhovsky YS, Krivovichev SV (2017). Barringerite Fe_2_P from pyrometamorphic rocks of the Hatrurim Formation, Israel. Geol. Ore Deposits.

[3] Britvin SN, Rudashevsky NS, Krivovichev SV, Burns PC, Polekhovsky YS (2002). Allabogdanite, (Fe,Ni)_2_P, a new mineral from the Onello meteorite: The occurrence and crystal structure. Am. Mineral..

[4] Britvin SN (2019). Allabogdanite, the high-pressure polymorph of (Fe,Ni)_2_P, a stishovite-grade indicator of impact processes in the Fe–Ni–P system. Sci. Rep..

[5] Buchwald, V. F. Handbook of iron meteorites, their history, distribution, composition and structure. University of California Press, Los Angeles, CA (1975).

[6] Chen, K., Jin, Z. & Peng, Z. The discovery of iron barriegerite (Fe_2_P) in China. *Dizhi Kexue*. 127–135 (in Chinese) (1983).

[7] Britvin SN (1999). Nickelphosphide (Ni,Fe)_3_P, the nickel analog of schreibersite. Zap. Ross. Mineral. O-va.

[8] Kopylova AG, Oleinikov BV, Sobolev NV, Sushko OA (1999). New iron meteorite Onello, a unique nickel-rich ataxite. Dokl. Acad. Sci..

[9] Kopylova A, Oleinikov B (2000). Phosphides and phosphorous sulfides of the Onello meteorite. Zap. Ross. Mineral. O-va.

[10] Litasov KD, Ishikawa A, Kopylova AG, Podgornykh NM, Pokhilenko NP (2019). Mineralogy, trace element composition, and classification of Onello high-Ni ataxite. Dokl. Earth Sci..

[11] Caron L (2013). Magnetocrystalline anisotropy and the magnetocaloric effect in Fe_2_P. Phys. Rev. B.

[12] Geng Y, Zhang Z, Tegus O, Dong C, Wang Y (2016). Microstructure, magnetic and magnetocaloric properties of Fe_2–x_Mn_x_P_0.4_Si_0.6_ alloys. Sci. China Mater..

[13] Fries M (2017). Microstructural and magnetic properties of Mn-Fe-P-Si (Fe_2_P-type) magnetocaloric compounds. Acta Mater..

[14] Gercsi Z (2013). Magnetoelastic effects in doped Fe_2_P. Phys. Rev. B.

[15] Hendricks SB, Kosting PR (1930). The Crystal Structure of Fe_2_P, Fe_2_N, Fe_3_N and FeB. Z. Kristallogr. Cryst. Mater..

[16] Carlsson B, Gölin M, Rundqvist S (1973). Determination of the homogeneity range and refinement of the crystal structure of Fe_2_P. J. Solid State Chem..

[17] Fujii H (1979). Polarized neutron diffraction study of Fe_2_P single crystal. J. Phys. Soc. Jpn..

[18] Senateur JP, Rouault A, Fruchart R, Capponi JJ, Perroux M (1976). Etude par spectrometrie Mossbauer des transformations cristallographiques sous hautes pressions de MnFeAs et Fe_2_P. Mater. Res. Bull..

[19] Dera P (2008). High‐pressure polymorphism of Fe_2_P and its implications for meteorites and Earth’s core. Geophys. Res. Lett..

[20] Holtstam D, Broman C, Söderhielm J, Zetterqvist A (2003). First discovery of stishovite in an iron meteorite. Meteorit. Planet. Sci..

[21] Litasov KD, Podgornykh NM (2017). Raman spectroscopy of various phosphate minerals and occurrence of tuite in the Elga IIE iron meteorite. J. Raman Spectrosc..

[22] Koumina A, Bacmann M, Fruchart D, Mesnaoui M, Wolfers P (2004). Crystal structure and magnetic properties of some MM’X pnictides investigated by neutron diffraction and magnetisation measurements. *Morocc*. J. Condens. Mat..

[23] Gu T, Wu X, Qin S, McCammon C, Dubrovinsky L (2013). Probing nonequivalent sites in iron phosphide Fe_2_P and its mechanism of phase transition. The Eur.Phys. J. B.

[24] Fruchart R, Roger A, Senateur JP (1969). Crystallographic and magnetic properties of solid solutions of the phosphides M_2_P, M = Cr, Mn, Fe, Co, and Ni. J. Appl. Phys..

[25] Ellner M, Mittemeijer EJ (2001). The reconstructive phase transformation β-Co_2_P → α-Co_2_P and the structure of the high-temperature phosphide β-Co_2_P. Z. Anorg. Allg. Chem..

[26] Sénateur JP (1973). La sélectivité des substitutions dans les phases MM’P étude de l’ordre par diffraction neutronique dans NiCoP. Mater. Res. Bull..

[27] Jernberg P, Yousif AA, Häggström L, Andersson Y (1984). A Mössbauer study of Fe_2_P_1−x_Si_x_ (x ≤ 0.35). J. Solid State Chem..

[28] Wu X, Qin S (2010). First-principles calculations of the structural stability of Fe_2_P. J. Phys: Conf. Ser..

[29] Bhat Soumya S, Gupta Kapil, Bhattacharjee Satadeep, Lee Seung-Cheol (2018). Role of zero-point effects in stabilizing the ground state structure of bulk Fe2P. Journal of Physics: Condensed Matter.

[30] Wyckoff R (1963). Manganese phosphide structure. Cryst. St..

[31] Britvin SN (2019). Murashkoite, FeP, a new terrestrial phosphide from pyrometamorphic rocks of the Hatrurim Formation, South Levant. Miner. Petrol..

[32] Lutterotti, L., Matthies, S. & Wenk, H.-R. MAUD (material analysis using diffraction): a user friendly Java program for Rietveld texture analysis and more. in Proceeding of the twelfth international conference on textures of materials (ICOTOM−12). 1599 (NRC Research Press Ottowa, Canada).

[33] Lu JF, Tsai CJ (2014). Hydrothermal phase transformation of hematite to magnetite. Nanoscale Res. Lett..

[34] Burba CM, Frech R (2006). Vibrational spectroscopic investigation of structurally-related LiFePO_4_, NaFePO_4_, and FePO_4_ compounds. Spectrochim. Acta Part A.

[35] Dera P (2009). Structure and behavior of the barringerite Ni end‐member, Ni_2_P, at deep Earth conditions and implications for natural Fe‐Ni phosphides in planetary cores. J. Geophys. Res. B: Solid Earth.

[36] Minin DA, Shatskiy AF, Litasov KD, Ohfuji H (2019). The Fe–Fe_2_P phase diagram at 6 GPa. High Pressure Res..

[37] Zhao Z (2017). Phase diagram, stability and electronic properties of an Fe–P system under high pressure: a first principles study. RSC Adv..

[38] McQueen TM (2008). Intrinsic properties of stoichiometric LaFePO. Phys. Rev. B.

[39] Singh DJ, Du M-H (2008). Density functional study of LaFeAsO_1−x_F_x_: A Low carrier density superconductor near itinerant magnetism. Phys. Rev. Lett..

[40] Litasov KD, Shatskiy A, Minin DA, Kuper KE, Ohfuji H (2019). The Ni-Ni_2_P phase diagram at 6 GPa with implication to meteorites and super-reduced terrestrial rocks. High Pressure Res..

[41] Litasov KD, Teplyakova SN, Shatskiy A, Kuper KE (2019). Fe-Ni-P-S melt pockets in Elga IIE iron meteorites: Evidence for the origin at high-pressures up to 20 GPa. Minerals.

[42] Kresse G, Furthmüller J (1996). Efficient iterative schemes for ab initio total-energy calculations using a plane-wave basis set. Phys. Rev. B.

[43] Kresse G, Joubert D (1999). From ultrasoft pseudopotentials to the projector augmented-wave method. Phys. Rev. B.

[44] Perdew JP, Burke K, Ernzerhof M (1996). Generalized gradient approximation made simple. Phys. Rev. Lett..

[45] Togo A, Tanaka I (2015). First principles phonon calculations in materials science. Scr. Mater..

