# Supplementary material for: (Fe,Ni)2P allabogdanite can be an ambient pressure phase in iron meteorites
Source: Sci Rep. 2020 Jun 2;10:8956. doi: 10.1038/s41598-020-66039-0 (PMC7265559; doi:10.1038/s41598-020-66039-0)
Supplement: Supplementary file 1 — Supplementary figures. [file 41598_2020_66039_MOESM1_ESM.docx]

***Scientific Reports***

**Supplementary materials for**

**(Fe,Ni)_2_P allabogdanite can be an ambient pressure phase in iron meteorites**

**Konstantin D. Litasov, Tatyana B. Bekker, Nursultan E. Sagatov,**

**Pavel N. Gavryushkin, Pavel G. Krinitsyn, Konstantin E. Kuper**


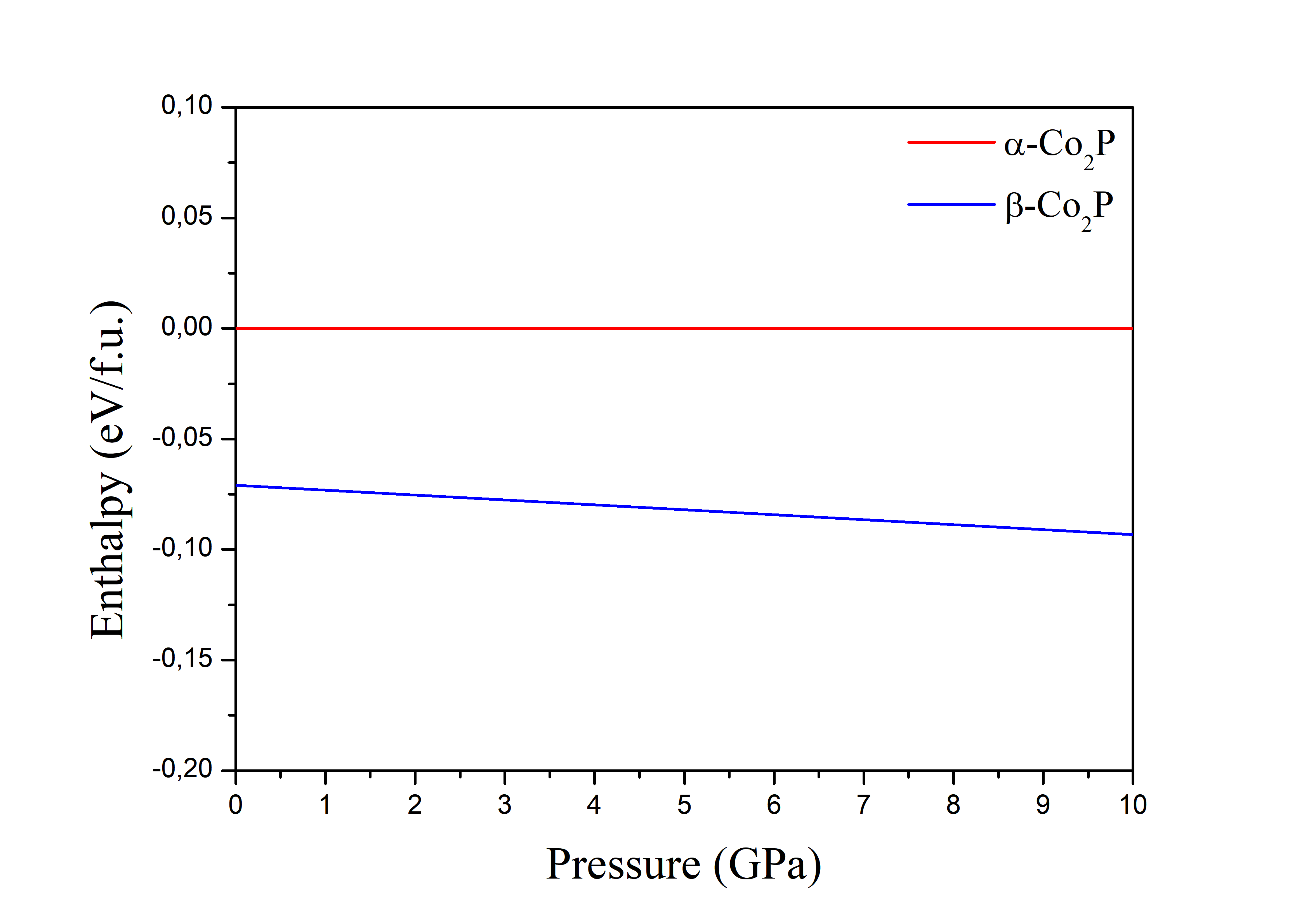


**Figure S1**. Dependence of enthalpy on pressure for the α-Co_2_P C22 hexagonal and β-Co_2_P C23 orthorombic phases.


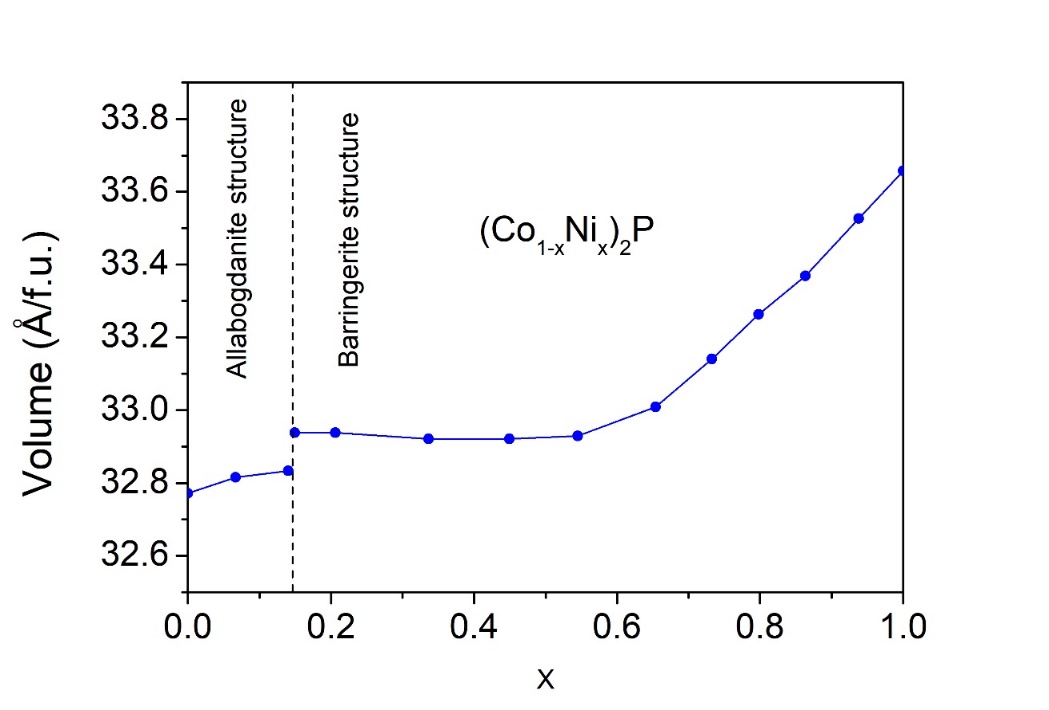


**Figure S2**. Phase transition in (Co_1-x_Ni_x_)_2_P solid solution (plotted based on Senateur et al.^26^).


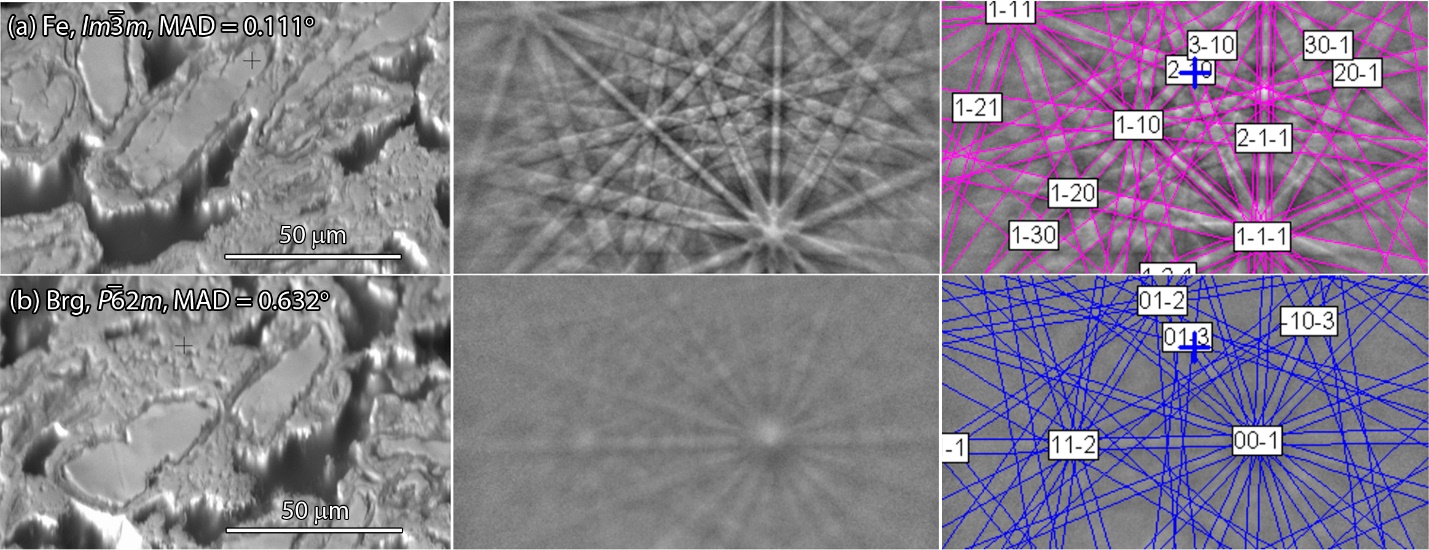


**Figure S3**. Electron backscatter diffraction from iron and barringerite (Brg) grain, sample (1.5Fe+0.5Ni+P), 773 K (Table 1). MAD – mean angular deviation.
